# Supplementary material for: A nigro–subthalamo–parabrachial pathway modulates pain-like behaviors
Source: Nat Commun. 2022 Dec 15;13:7756. doi: 10.1038/s41467-022-35474-0 (PMC9755217; doi:10.1038/s41467-022-35474-0)
Supplement: Supplementary file 1 — Supplementary Information [file 41467_2022_35474_MOESM1_ESM.pdf]

Supplementary Information

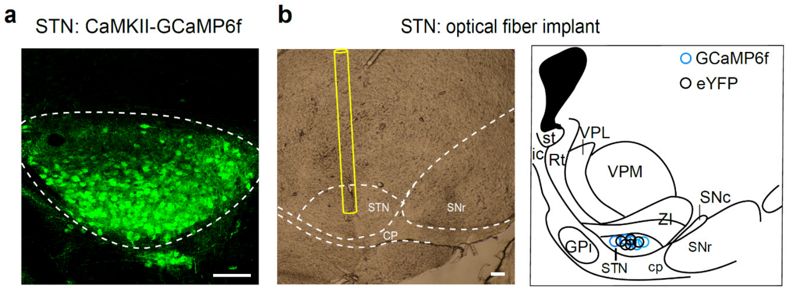

Supplementary figure 1 locations of virus injections and optical fiber implants in the STN for GCaMP6f signal recordings. (a) Example image of GCaMP6f expression in the STN. **(b)** Example image (left, from 5 experiments) and schematic diagram (right) of locations of optical fiber implants in the STN for GCaMP6f signal recordings. Open circles indicate the locations of optical fiber implants. Scale bar, 100 µm.

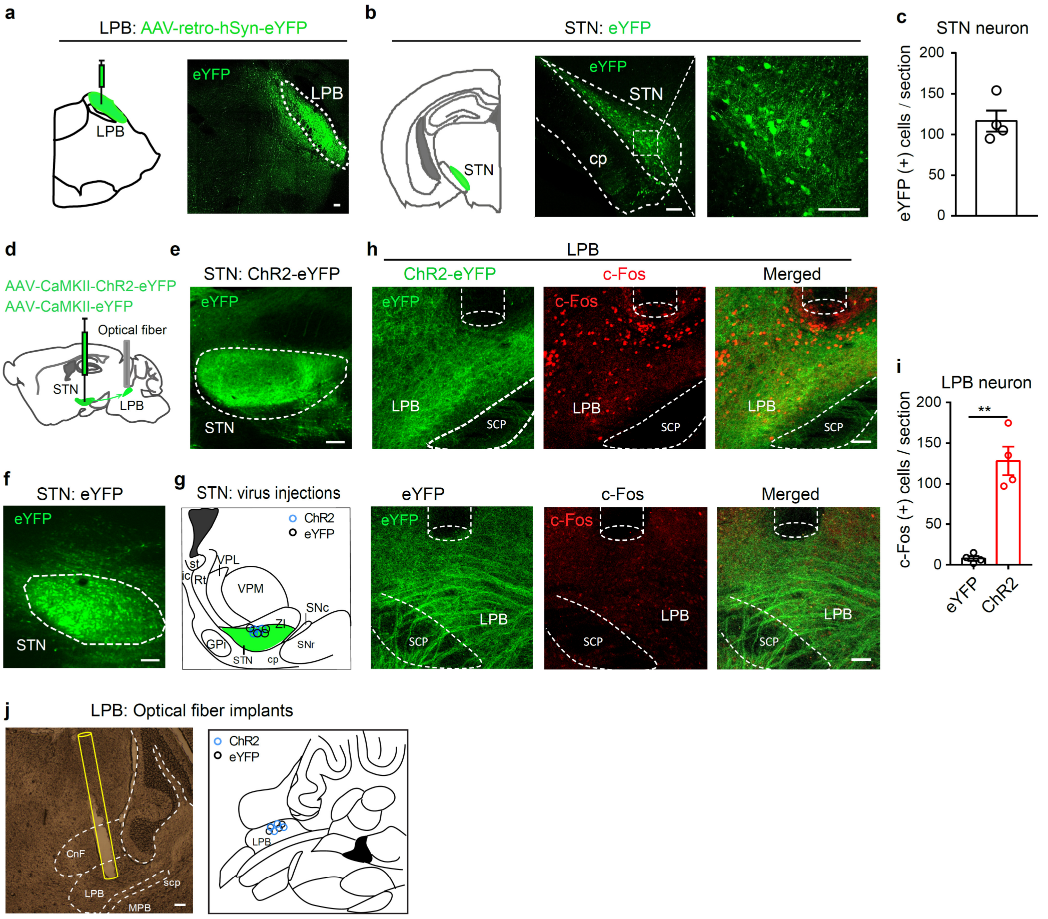

Supplementary figure 2 Optogenetic excitation of the STN–LPB projection increases c-Fos expression in the LPB. (a) Schematic diagram and example image (from 4 experiments) of retrograde virus injection in the LPB. **(b)** AAV-retro viral expression in the STN and example image (from 4 experiments) of eYFP-labeled STN neurons. **(c)** Bar graph showing quantification of eYFP-labeled STN neurons (116.5 ± 12.98 cells per section) from 4 mice. **(d)** Schematic diagram of optogenetic manipulation of the STN–LPB projection with ChR2-eYFP or eYFP injection into the STN and optical fiber implantation in the LPB. **(e - f)** Example images showing ChR2-eYFP or eYFP expression in the STN. **(g)** Schematic diagram of locations of virus injections in the STN for panels (**e - f**). **(h)** Example images (from 4 experiments) of c-Fos expression in the LPB after optogenetic stimulation (473 nm, 5 ms, 20 Hz, 4 mW, 2 min light with 2 min interval for 30 min) of ChR2-eYFP (upper panels) or eYFP (lower panels) labeled STN axonal terminals in the LPB. **(i)** Bar graph showing quantification of c-Fos (+) neurons in the LPB following blue light illumination (t =

6.71, P = 0.0005, n = 4 mice each group, 5 LPB sections from each mouse were counted and averaged). **(j)** Example image (left) and schematic diagram (right) of locations of optical fiber implants in the LPB. ** P < 0.01, two-tailed *t*-test for **(i)**. All data are presented as mean ± SEM. all scale bars: 100 μm.

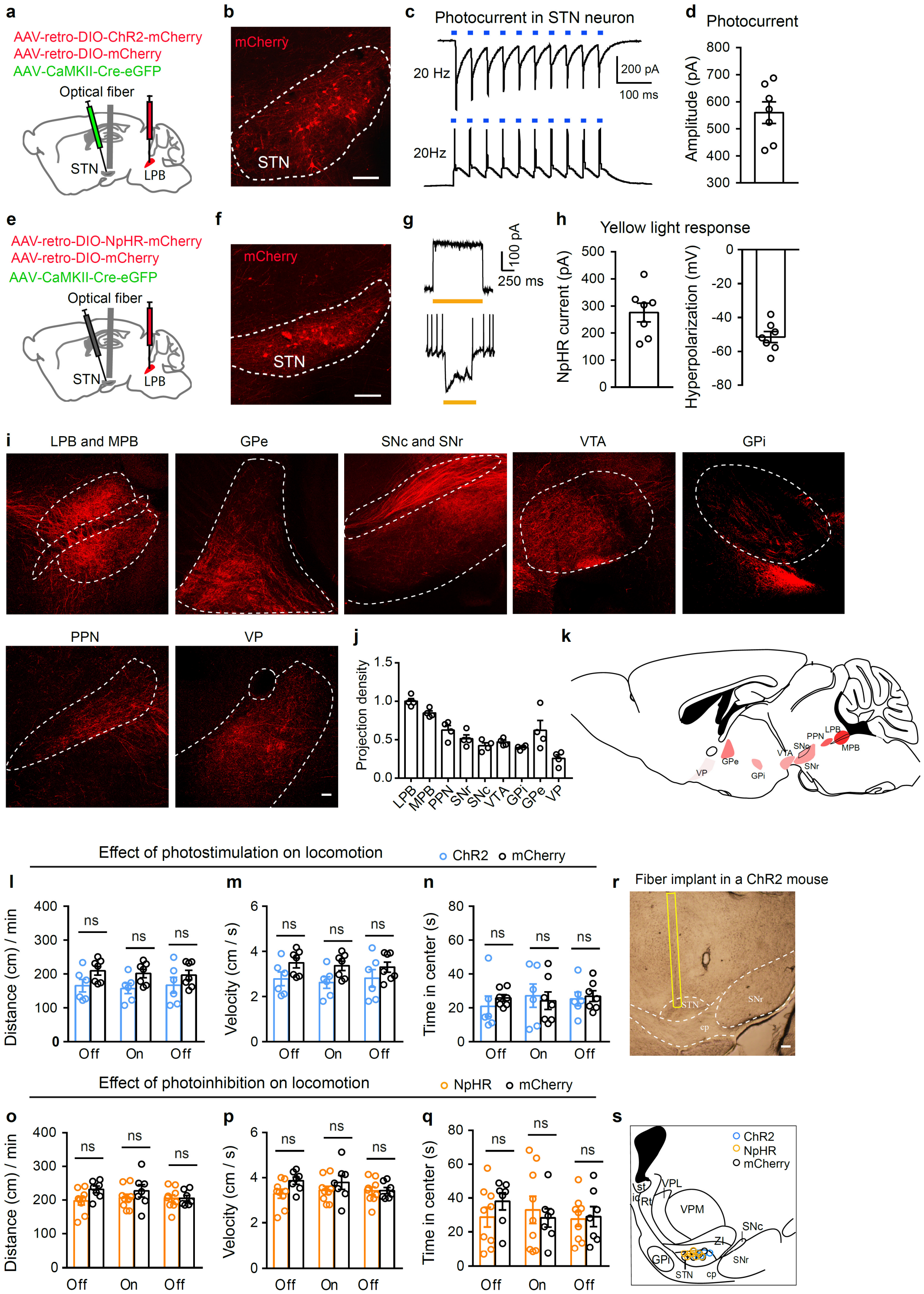

Supplementary figure 3 Optogenetic modulation of STN-LPB neurons does not affect locomotion. (a) Diagram of virus injections and optical fiber implants for optogenetic activation of STN-LPB neurons. **(b)** Example image (from 7 experiments) of AAV-retro-DIO-ChR2-mCherry in Cre(+) STN neurons projecting to the LPB. **(c - d)** Example trace (**c**, upper) and quantification **(d)** of corresponding inward currents

and firing (**c**, lower) recorded in an STN neuron with 20 Hz blue light stimulation. n = 7 cells from mice in **(d)**. **(e)** Diagram of virus injections and optical fiber implants for optogenetic silencing of STN-LPB neurons. **(f)** Example image (from 7 experiments) of AAV-retro-DIO-NpHR-mCherry in Cre (+) STN neurons projecting to the LPB. **(g - h)** Example traces **(g)** and quantification **(h)** of the corresponding outward current (g, upper) and hyperpolarization of membrane potential (g, lower) in response to yellow light stimulation. n = 8 neurons from 4 mice in **(h)**. **(i - j)** Downstream nuclei of STN-LPB neurons. Target structures (i, from 4 experiments) and quantification **(j)** of relative fluorescence density of mCherry-labeled processes. **(k)** Mapping of major brain regions receiving axonal outputs of LPB-projecting STN neurons. n = 4 mice. LPB: lateral parabrachial nucleus; MPB: medial parabrachial nucleus; PPN: pedunculopontine nucleus; SNr: substantia nigra pars reticulata; SNc: substantia nigra pars compacta; VTA: ventral tegmental area; GPi and GPe: Internal and external segments of globus pallidus; VP: ventral pallidum. **(l - q)** Effect of activation ($n \geq 6$ per group) or inhibition ($n \geq 9$ per group) of STN-LPB neurons on locomotion in the open field test. **(l)** $F_{(2, 22)} = 0.67$, $P = 0.52$. **(m)** $F_{(2, 22)} = 0.67$, $P = 0.52$. **(n)** $F_{(2, 22)} = 0.33$, $P = 0.72$. **(o)** $F_{(2, 28)} = 3.29$, $P = 0.06$. **(p)** $F_{(2,28)} = 3.29$, $P = 0.06$. **(q)** $F_{(2, 28)} = 0.84$, $P = 0.44$. **(r - s)** Example image **(r)** and diagram **(s)** of locations of optical implants in the STN. Open circles indicate locations of optical implants. Two-way ANOVA with Tukey's post-hoc analysis for **(l - q)**. Data are presented as mean ± SEM. Scale bars: 100 μm.

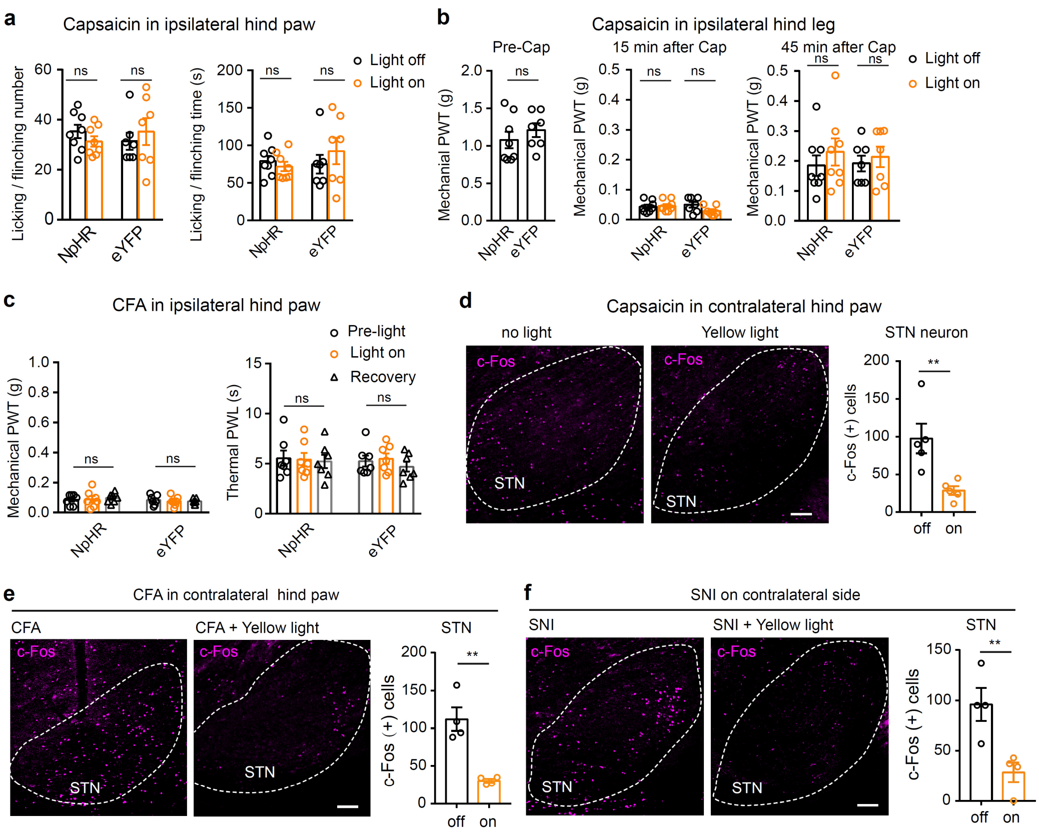

Supplementary figure 4 Optogenetic silencing of STN-LPB neurons does not affect ipsilateral pain processing but attenuates the hyperactivity of STN neurons in contralateral pain states. (a) Effect of optogenetic silencing of STN-LPB neurons on the frequency (Left, $F_{(1, 13)} = 1.2$, $P = 0.27$) and duration ($F_{(1, 13)} = 1.2$, $P = 0.29$) of licking / flinching behavior during a 15-min testing period after capsaicin injection into ipsilateral hind paws. n ≥ 7 per group. **(b)** Effect of optogenetic silencing of STN-LPB neurons on capsaicin-induced secondary mechanical allodynia on the ipsilateral hind paw. Left: mechanical threshold in NpHR and eYFP mice ($t = 0.89$, $P = 0.39$); middle and right: PWT tested during 15 – 30 min ($F_{(1, 13)} = 2.6$, $P = 0.13$) and 45 – 60 min ($F_{(1, 13)} = 0.39$, $P = 0.54$) after subcutaneous capsaicin injection in the ipsilateral hind leg. n ≥ 7 per group. **(c)** Effect of optogenetic silencing of LPB-projecting STN neurons on PWT ($F_{(2, 24)} = 0.37$, $P =0.69$) and PWL ($F_{(2, 24)} = 0.45$, $P = 0.64$) 24 h after CFA injection in the ipsilateral hind paws. **(d - f)** Example images (from 5 experiments in panel d, from 4 experiments in panel e, from 4 experiments in panel f) and quantification of c-Fos (+) neurons in the STN in the capsaicin (Cap), CFA, and SNI mice depicted in

Figure 4 with or without yellow-light illumination. **(d)** Right, t = 3.39, P = 0.0095, n = 5 mice. **(e)** Right, t = 5.16, P = 0.002, n = 4 mice. **(f)** Right, t = 3.55, P = 0.01, n = 4 mice. ** $P < 0.01$; Two-way ANOVA with Tukey's post-hoc analysis for **(a)**, (**b**, middle and right panel) and **(c)**. Two-tailed unpaired *t*-test for (**b**, left panel), **(d)**, **(e)** and **(f)**. Data are presented as mean ± SEM. Scale bars: 100 μm.

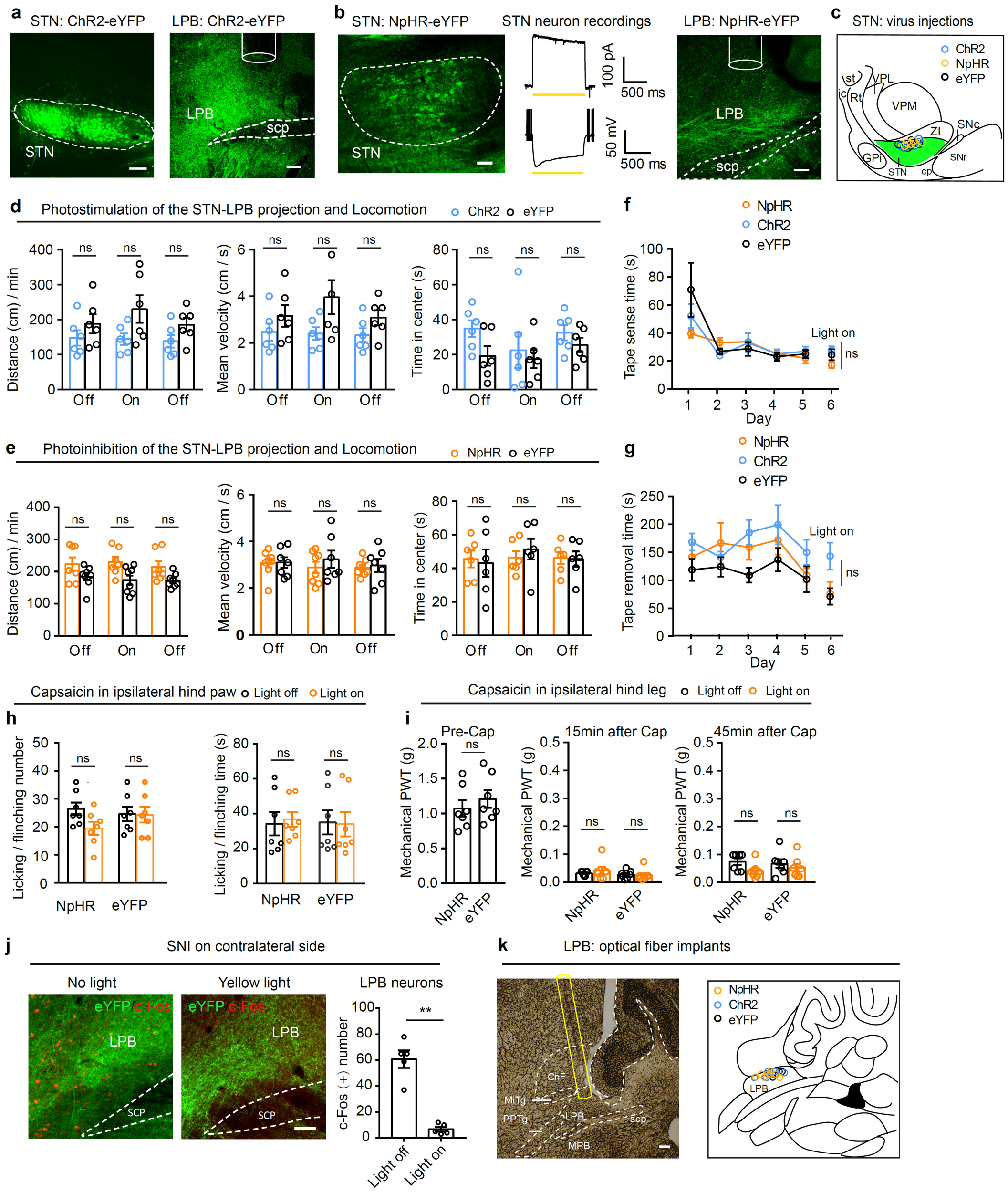

Supplementary figure 5 Optogenetic modulation of the STN–LPB projection does not affect ipsilateral pain thresholds and locomotion. (a - b) Example images of ChR2 (from 7 experiments) and NpHR (from 6 experiments) expression in the STN and ChR2-labeled terminals in the LPB. **(b)** Middle: yellow-light-evoked outward current and hyperpolarization in an NpHR-expressing STN neuron. **(c)** Diagram showing locations of virus injections. **(d - g)** Effect of blue or yellow light on the STN-LPB projection on motor skills (n ≥ 6 per group). **(d)** Left: $F_{(2, 22)} = 1.7$, $P = 0.20$; Middle: $F_{(2, 22)} = 2.0$, $P = 0.16$; Right: $F_{(2, 22)} = 0.54$, $P = 0.59$. **(e)** Left: $F_{(2, 26)} = 0.46$, $P = 0.64$; Middle: $F_{(2, 26)} = 0.46$, $P = 0.63$; Right: $F_{(2, 26)} = 0.57$, $P = 0.57$. **(f)** $F_{(10, 105)} = 1.86$, P =

0.06. **(g)** $F_{(10, 105)} = 0.93$, $P = 0.51$. Blue bars in **(f, g)** indicate blue or yellow light to activate ChR2 or NpHR in the LPB. **(h - i)** Effect of silencing of the STN–LPB projection on capsaicin-induced nocifensive behavior and secondary mechanical hyperalgesia (n = 7). **(h)** Left, $F_{(1, 12)} = 3.18$，$P = 0.1$; Right, $F_{(1, 12)} = 0.77$，$P = 0.39$. **(i)** Left: $t = 0.78$, $P = 0.45$; Middle: $F_{(1, 12)} = 0.55$, $P = 0.47$; Right: $F_{(1, 12)} = 0.82$，$P = 0.38$. **(j)** Example images (from 5 experiments) and quantification of c-Fos(+) LPB neurons ($t = 7.78$, $P < 0.0001$; n = 5). **(k)** Example image and diagram of locations of optical fiber implants in the LPB. ** $P < 0.01$; Two-way ANOVA with Tukey's post-hoc analysis for **(d - h)** and (**i**, middle and right panel); Two-tailed unpaired *t*-test for (**i**, left panel) and (**j**, right panel). Open circles in **(c)** and **(k)** indicate locations of virus injection or optical fiber implant. Data are presented as mean ± SEM. Scale bars: 100 μm.

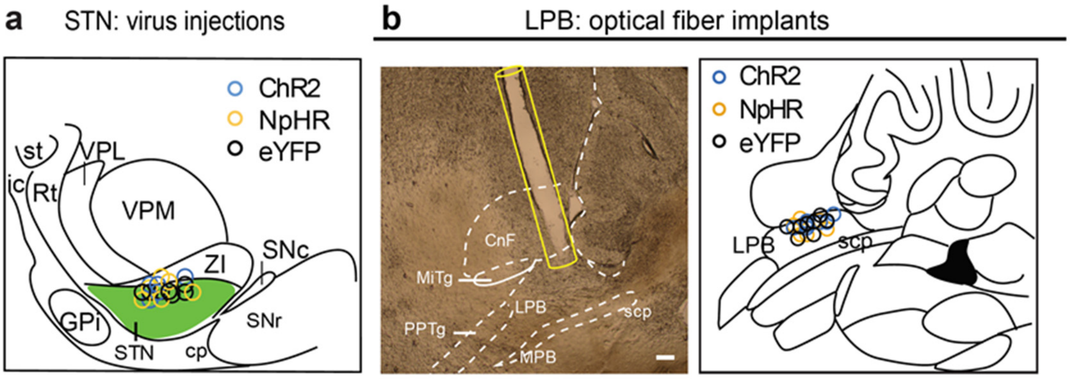

Supplementary figure 6 Locations of virus injections and optical fiber implants in female mice. (a) Diagram showing virus injection sites in the STN. **(b)** Locations of optical fiber implants in the LPB. Open circles in panel indicate sites of virus injections or optical fiber implants. Scale bars: 100 μm.

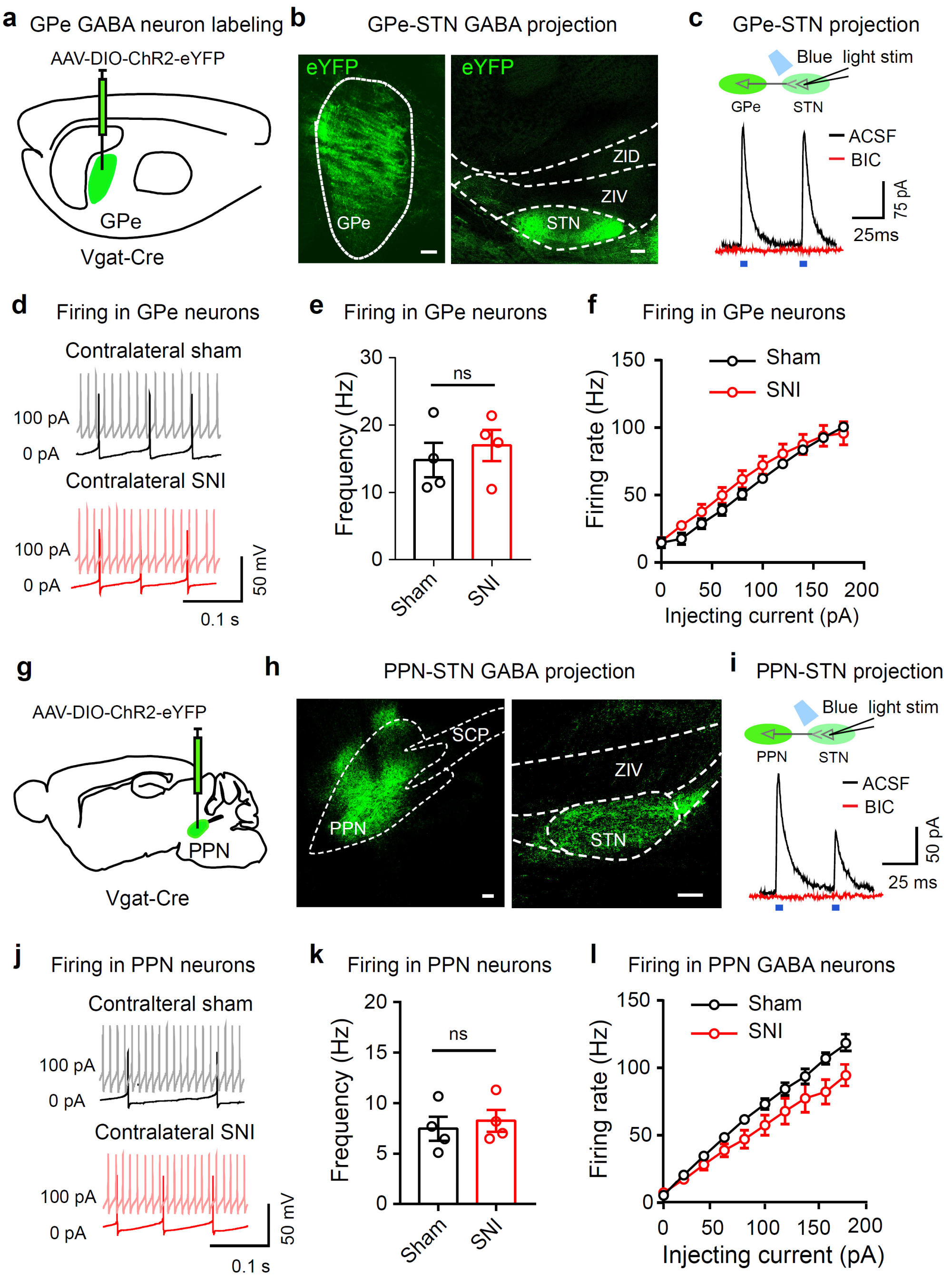

Supplementary figure 7 SNI does not affect the excitability of GABA neurons in the GPe and PPN. **(a)** Schematic diagram of labeling of GPe^{GABA} neurons. **(b)** Example images (from 4 experiments) showing ChR2 expression in the GPe (left) and ChR2-labeled terminals in the STN (right). **(c)** Whole-cell patch clamp recording of blue light-evoked currents in STN neurons (top). Photo-currents in STN neurons (bottom) were blocked by 10 μM BIC. **(d)** Firing recorded from GPe^{GABA} neurons of sham and SNI mice. **(e - f)** Summary of spontaneous **(e)** and evoked **(f)** firing recorded

from ChR2-labeled GPe^{GABA} neurons in sham and SNI mice. **(e)** $t = 0.63$, $P = 0.55$, $n = 4$ per group. **(f)** $F_{(9, 99)} = 0.57$, $P = 0.82$; $n = 3$ mice per group. **(g)** Schematic diagram of labeling of PPN^{GABA} neurons. **(h)** Example images (from 4 experiments) showing expression of ChR2 in the PPN (left) and ChR2-labeled terminals in the STN (right). **(i)** Whole-cell patch-clamp recording of blue-light-evoked currents in STN neurons. Blue-light-evoked currents were BIC-sensitive (bottom). **(j)** Firing recorded from PPN^{GABA} neurons from sham and SNI mice. **(k - l)** Summary of spontaneous **(k)** and evoked **(l)** firing recorded from PPN^{GABA} neurons in sham and SNI mice. **(k)** $t = 0.48$, $P = 0.65$, $n = 4$ mice per group. **(l)** $F_{(9, 80)} = 1.03$, $P = 0.42$; $n = 3$ mice per group. Two-tailed *t*-test for **(e)** and **(k)**; Two-way ANOVA with Turkey's post-hoc analysis for **(f)** and **(l)**. Data are presented as mean ± SEM. Scale bars: 100 μm.

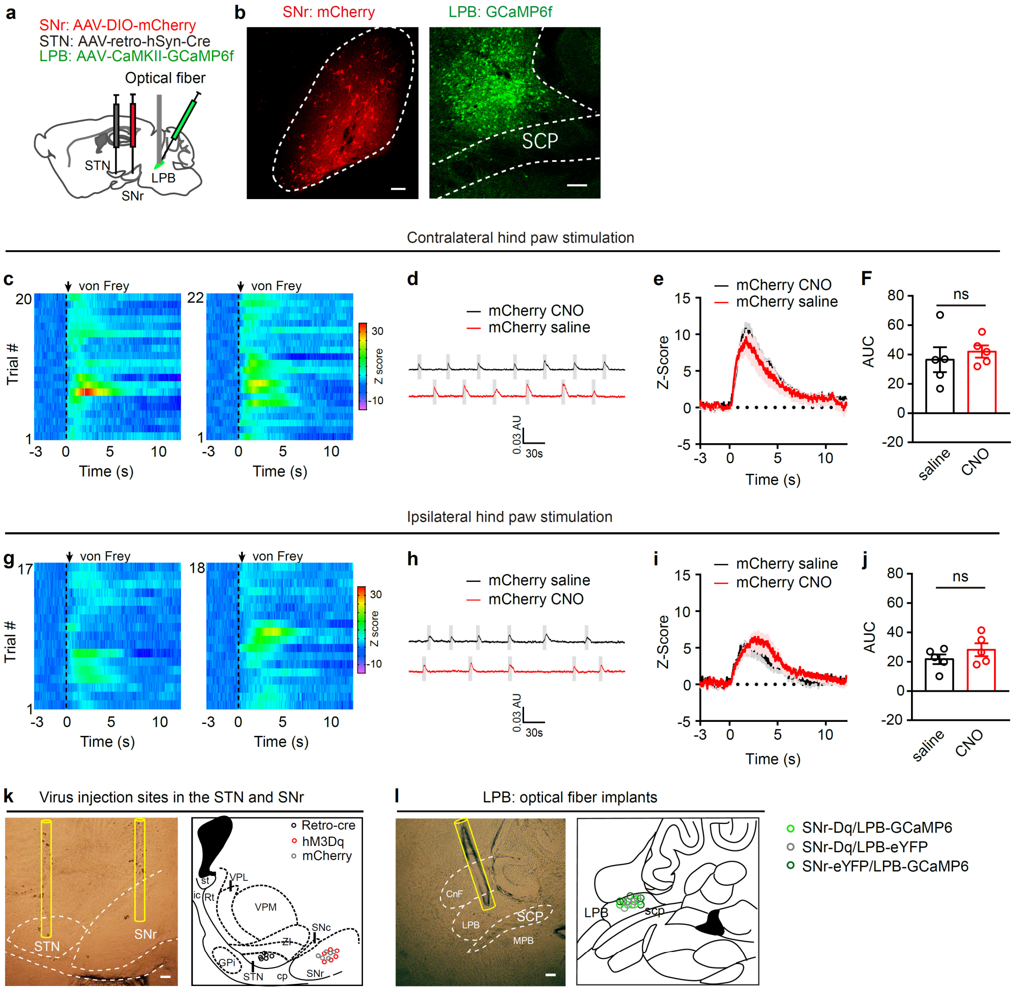

Supplementary figure 8 Connectivity of the SNr^{GABA}–STN^{Glu}–LPB^{Glu} pathway. (a) Schematic diagram for measuring the response of LPB^{Glu} neurons to mechanical stimulation with saline or CNO administration. **(b)** Example images (from 5 experiments) showing mCherry-labeled SNr neurons and GCaMP6f expressing LPB neurons. **(c - j)** GCaMP6f signal was recorded with fiber photometry before, during, and after von Frey fiber (4 g) stimulation on hind paws. CNO (i.p., 3 mg/kg) or saline (i.p.) was applied 45 min prior to GCaMP6f signal recording. Heat maps (**c** and **g**), example traces (**d** and **h**), average traces (**e** and **i**), and quantification (**f** and **j**, AUC in panels **e** and **i**) of GCaMP6f response in the LPB of mice receiving von Frey stimulation of the contralateral or ipsilateral hind paw after saline or CNO administration. **(f)** t = 0.93, P = 0.4. **(j)** t = 0.98, P = 0.38; Two-tailed paired t test; n = 5 mice. **(k - l)** Example images (left) and diagrams (right) of locations of virus injection and optical fiber implants (Open circles indicate injection sites or the locations of optical fiber implants).

AU in panels **(d)** and **(h)** stands for arbitrary unit of fluorescence intensity. Data are presented as mean ± SEM. Scale bars: 100 μm.

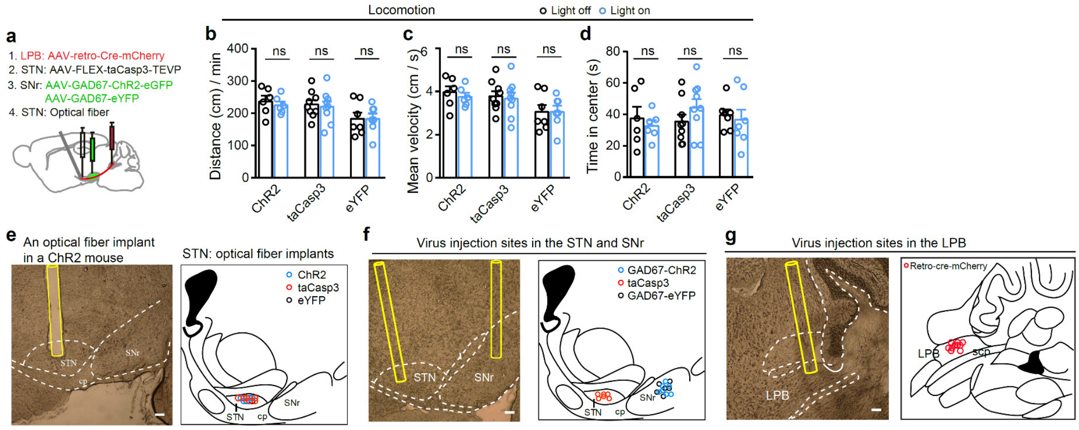

Supplementary figure 9 Optogenetic activation of the SNr^{GABA}–STN^{Glu}–LPB^{Glu} pathway does not change locomotion in physiological condition. (a) Schematic diagram for taCasp3-mediated ablation of STN–LPB neurons and optogenetic activation of axonal terminals of SNr GABA neurons in the STN. **(b - d)** Effect of disruption of the SNr–STN–LPB pathway on locomotion in the open field test. **(b)** $F_{(2, 39)} = 0.51$, $P = 0.6$. **(c)** $F_{(2, 39)} = 0.51$, $P = 0.6$. **(d)** $F_{(2, 39)} = 1.05$, $P = 0.41$. **(e)** Example image (left) and diagram (right) of locations of optical fiber implants in the STN (Open circles indicate the locations of optical fiber implants). **(f - g)** Example images (left) and diagrams (right) of locations of virus injection into the STN, SNr, and LPB (Open circles indicate the injection sites). Two-way ANOVA with Turkey's post-hoc analysis for **(b - d)**. Scale bars: 100 µm. Data are presented as mean ± SEM. ChR2: SNr-ChR2, STN-mCherry; taCasp3: SNr-ChR2, STN-taCasp3 lesion; eYFP: SNr-eYFP, STN-mCherry.

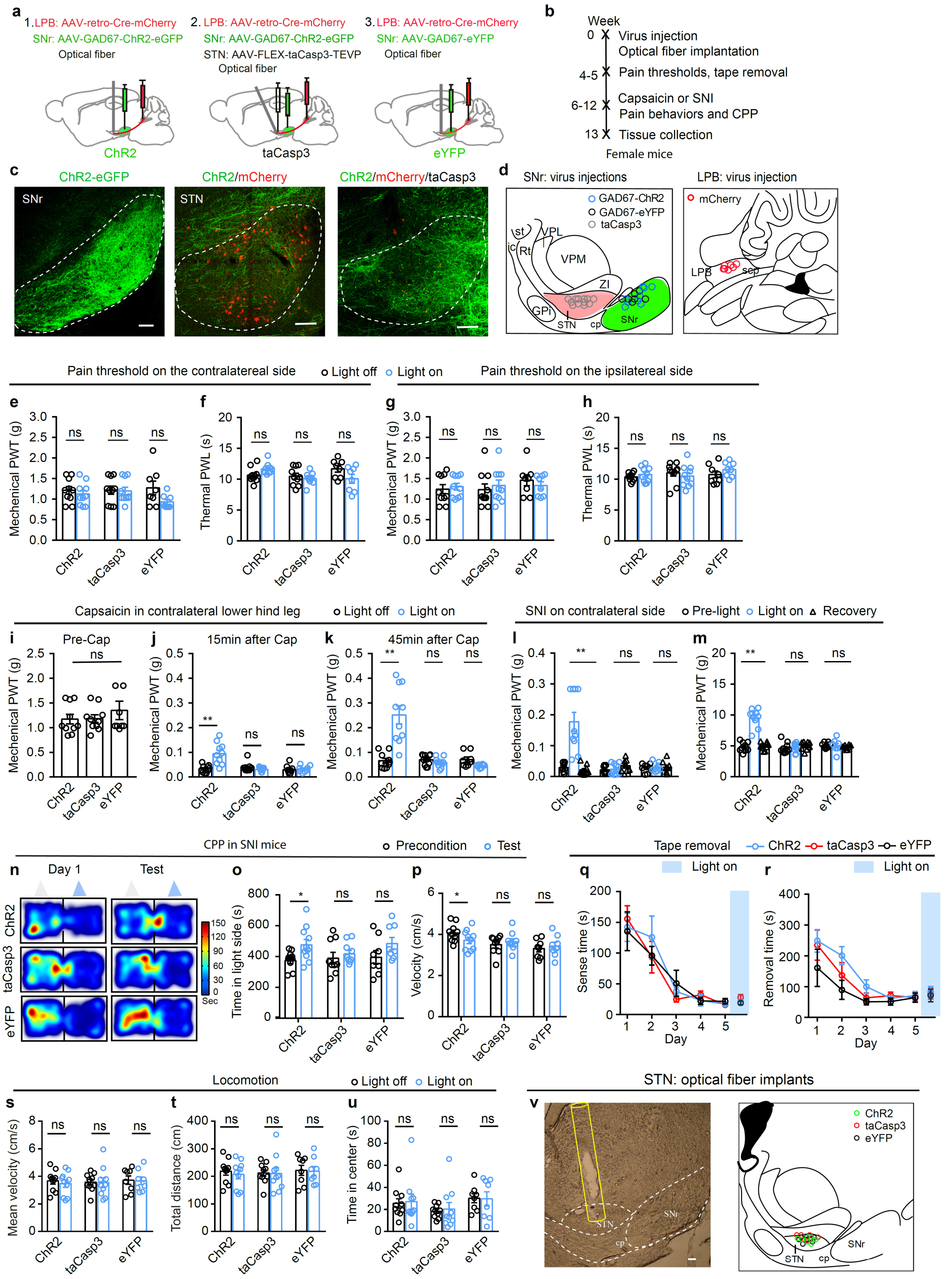

Supplementary figure 10 The SNr^{GABA}–STN^{Glu}–LPB^{Glu} pathway in pain modulation in female mice. (a - b) Schematic diagram and timeline of experimental setup. **(c)** Example images of virus expression (from 5 experiments). **(d)** Virus injection sites in the STN, SNr and LPB. **(e - h)** Effect of disruption of the SNr–STN–LPB pathway on PWT and PWL of the hind paws. **(e)** $F_{(1, 25)} = 3.14$, $P = 0.088$. **(f)** $F_{(1, 25)} =$

0.02, P = 0.9. **(g)** $F_{(1, 25)} = 76$, P = 0.39. **(h)** $F_{(1, 25)} = 0.5$, P = 0.48. n ≥ 8 each group. **(i - m)** Effect of disruption of SNr-STN-LPB pathway on pain thresholds in Cap and SNI mice. **(i)** $F_{(, 25)} = 0.62$, P = 0.66. **(j)** (F $_{(1, 26)}$ = 9.5, P = 0.0048. **(k)** $F_{(1, 26)} = 11.7$, P = 0.0021. **(l)** F $_{(2, 50)}$ = 18.29, P < 0.0001. **(m)** $F_{(2, 50)} = 44.06$, P < 0.0001. n ≥ 8 per group. **(n - p)** Representative heat maps **(n)** and time spent **(o)** and velocity **(p)** in the blue-light-paired chamber in pre-test and test session in SNI mice (n ≥ 8 per group). Grey and blue triangles in panel **(n)** represent no light and blue light presented in the chamber during the conditioning session, respectively. **(o)** $F_{(1, 24)} = 9.91$, P = 0.004. **(p)** $F_{(1, 24)} = 0.15$, P = 0.71. **(q - u)** disruption of the SNr–STN–LPB pathway had no effect on motor skill (n ≥ 8 per group). **(q)** $F_{(2, 25)} = 0.081$, P = 0.92; Light off vs on: $F_{(5, 50)} = 0.82$, P = 0.54. **(r)** $F_{(2, 25)} = 2.27$, P = 0.12; Light off vs on: $F_{(5, 50)} = 0.39$, P = 0.85. **(s)** $F_{(1, 25)} = 0.39$, P = 0.54. **(t)** $F_{(1, 25)} = 0.35$, P = 0.56. **(u)** $F_{(1, 25)} = 0.06$, P = 0.8. **(v)** Locations of optical fiber implants in the STN. Open circles in panel **(d)** and **(v)** indicate the virus injection sites and locations of optical fiber implants, respectively. * P < 0.05, ** P < 0.01; One-way ANOVA with Tukey's post-hoc analysis for panel **(i)**; Two-way ANOVA with Tukey's post-hoc analysis for **(e - h)**, **(j – m),** and **(o - u)**. Data are presented as mean ± SEM. Scale bars: 100 μm.

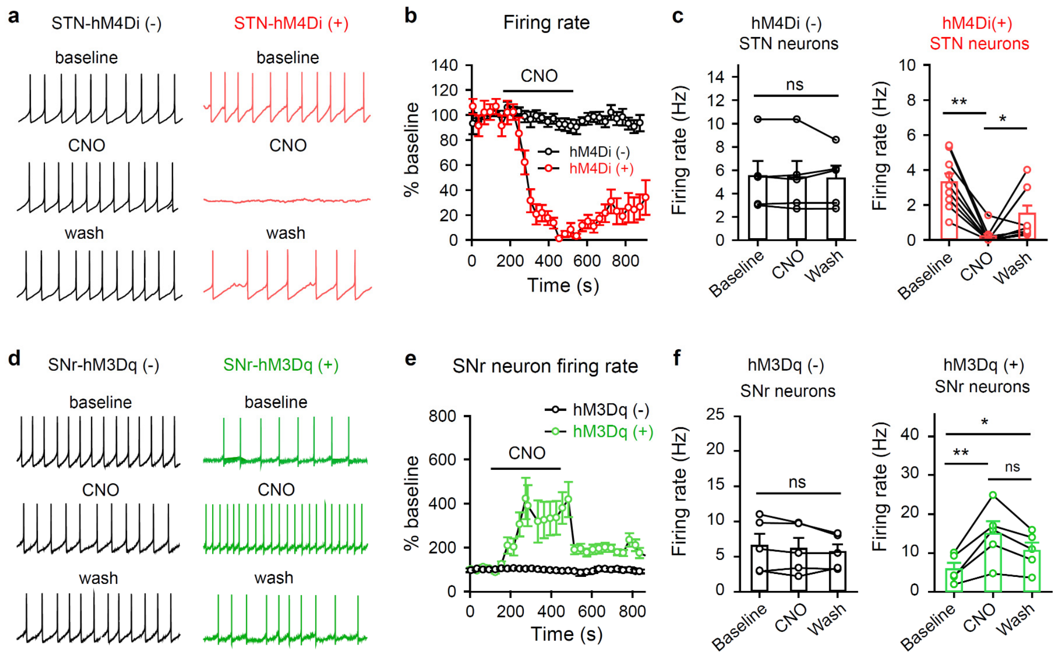

Supplementary figure 11 Chemogenetic manipulation of the firing of STN and SNr neurons. Whole-cell patch-clamp recordings were performed on STN and SNr neurons from wild type mice without viral injection (hM4Di(-) or hM3Dq(-)), on hM4Di(+) STN neurons from mice received AAV-CaMKII-hM4Di-mCherry injection in the STN, on hM3Dq(+) SNr neurons from Vgat-Cre mice received AAV-EF1α-DIO-hM3Dq-mCherry injection in the SNr. **(a)** Representative traces (2 s) of firing from an STN neuron from hM4Di(-) and hM4Di(+) mice before (baseline), during (CNO), and after (wash) bath application of 3 μM CNO. **(b)** Time courses of the effect of CNO on firing rate in hM4Di(-) and hM4Di(+) STN neurons. **(c)** CNO did not affect firing rate of STN neurons from hM4Di (-) mice (Left panel: $F = 0.08$, $P = 0.92$, One way repeated measures ANOVA), but inhibited firing rate of hM4Di(+) STN neurons (Right panel: $F = 16.17$, $P < 0.001$, One way repeated measures ANOVA). Data in **(b)** and **(c)** were from 5 hM4Di (-) mice and 5 hM4Di (+) mice. **(d)** Representative traces (2 s) of firing from hM3Dq (-) and hM3Dq (+) SNr neurons before (baseline), during (CNO), and after (wash) bath application of 3 μM CNO. **(e)** Time courses of the effect of CNO on firing rate of hM3Dq(-) and hM3Dq (+) STN neurons. **(f)** CNO did not affect firing rate of STN neurons from hM3Dq (-) mice (Left panel: $F = 1.50$, $P = 0.28$, One way repeated measures ANOVA), but enhanced

firing rate of hM3Dq (+) STN neurons (Right panel: $F = 18.51$, $P < 0.001$, One way repeated measures ANOVA). Data in **(e)** and **(f)** were from 5 WT mice and 5 hM3Dq (+) mice. Data are presented as mean ± SEM.

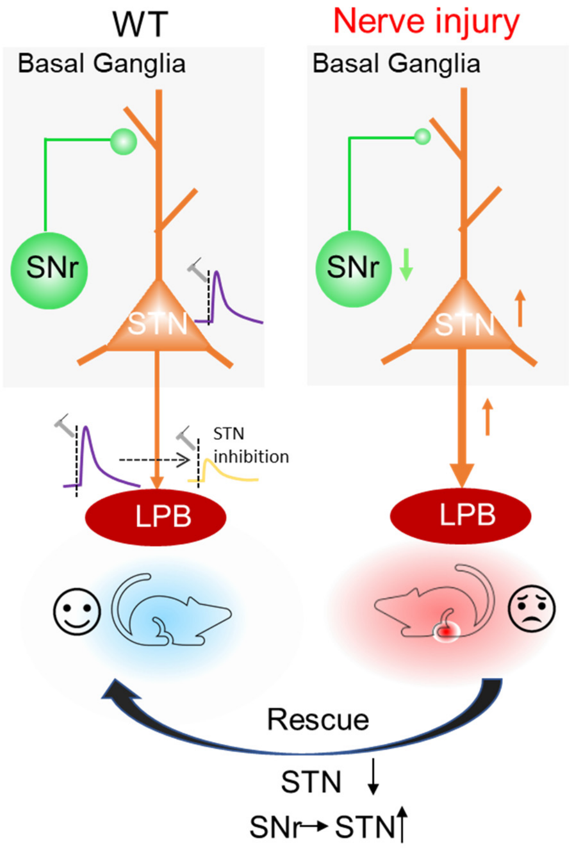

Supplementary figure 12 Neuroplasticity in SNr^{GABA}–STN^{Glu}–LPB^{Glu} pathway after nerve injury. **Left**: In the SNr^{GABA}-STN^{Glu}-LPB^{Glu} pathway, both STN^{Glu} and LPB^{Glu} neurons respond to peripheral nociceptive stimulation. Inhibition of STN^{Glu} neurons or stimulation of SNr^{GABA} neurons attenuates nociceptive responses in LPB neurons, mitigating pain-like behavior and spontaneous pain during pain states.

Right: After peripheral nerve injury, SNr^{GABA} neurons become hypoactive, but STN^{Glu} and LPB^{Glu} neurons are hyperactive and the STN^{Glu}-LPB^{Glu} projection is strengthened. These long-term alterations may be related to hypersensitivity to nociceptive stimuli. Reversing the dysfunction in the SNr-STN-LPB pathway by either optogenetic inhibition of STN neurons or optogenetic activation of SNr-STN GABAergic projection partially relieves hypersensitivity to nociceptive stimuli and aversion.

Supplementary Table 1: The resting membrane potential (Rm) and input resistance (Ir) of STN glutamatergic neurons and LPB glutamatergic neurons recorded in sham and SNI mice

	STN glutamatergic neurons		LPB glutamatergic neurons	
	Rm	Ir	Rm	Ir
Sham	-50.2 ± 1.1, n=5	979.1 ± 48.1, n=5	-50.9 ± 1.2, n=9	932.7 ± 67.8, n=9
SNI	-49.1 ± 1.0, n=5	1054 ± 50.6, n=5	-48.8 ± 1.1, n=9	830.8 ± 72.4, n=9

n represents the number of animals.

Supplementary Table 2: The resting membrane potential (Rm) and input resistance (Ir) in neurons recorded in sham and SNI mice

	SNr GABA neurons		GPe GABA neurons		PPN GABA neurons	
	Rm	Ir	Rm	Ir	Rm	Ir
Sham	-50.4 ± 0.8, n=6	606.4 ± 47.8, n=6	-48.4 ± 1.4, n=4	647.3 ± 72.6, n=4	-49 ± 1.2, n=4	912 ± 84.4, n=4
SNI	-50.7 ± 0.8, n=6	769.5 ± 72.9, n=6	-50.7 ± 1.7, n=4	853.7 ± 90.6, n=4	-45.9 ± 1.4, n=4	868.9 ± 94.2, n=4

n represents the number of animals.
