# Supplementary material for: A nigro–subthalamo–parabrachial pathway modulates pain-like behaviors
Source: Nat Commun. 2022 Dec 15;13:7756. doi: 10.1038/s41467-022-35474-0 (PMC9755217; doi:10.1038/s41467-022-35474-0)
Supplement: Supplementary file 3 — Reporting Summary [file 41467_2022_35474_MOESM3_ESM.pdf]

nature portfolio

Corresponding author(s): Chunyi Zhou, Cheng Xiao, Jun-Li Cao

Last updated by author(s): Nov 7, 2022

Reporting Summary

Nature Portfolio wishes to improve the reproducibility of the work that we publish. This form provides structure for consistency and transparency in reporting. For further information on Nature Portfolio policies, see our Editorial Policies and the Editorial Policy Checklist.

Statistics

For all statistical analyses, confirm that the following items are present in the figure legend, table legend, main text, or Methods section.

n/a	Confirmed	
☐	☒	The exact sample size (n) for each experimental group/condition, given as a discrete number and unit of measurement
☐	☒	A statement on whether measurements were taken from distinct samples or whether the same sample was measured repeatedly
☐	☒	The statistical test(s) used AND whether they are one- or two-sided<br>*Only common tests should be described solely by name; describe more complex techniques in the Methods section.*
☒	☐	A description of all covariates tested
☐	☒	A description of any assumptions or corrections, such as tests of normality and adjustment for multiple comparisons
☐	☒	A full description of the statistical parameters including central tendency (e.g. means) or other basic estimates (e.g. regression coefficient) AND variation (e.g. standard deviation) or associated estimates of uncertainty (e.g. confidence intervals)
☐	☒	For null hypothesis testing, the test statistic (e.g. F, t, r) with confidence intervals, effect sizes, degrees of freedom and P value noted<br>*Give P values as exact values whenever suitable.*
☒	☐	For Bayesian analysis, information on the choice of priors and Markov chain Monte Carlo settings
☒	☐	For hierarchical and complex designs, identification of the appropriate level for tests and full reporting of outcomes
☒	☐	Estimates of effect sizes (e.g. Cohen's d, Pearson's r), indicating how they were calculated

Our web collection on statistics for biologists contains articles on many of the points above.

Software and code

Policy information about availability of computer code

Data collection	1. Mouse behaviors were recorded through an infra-red camera controlled by an animal tracking software, Ethovision 11.5 (Noldus), and saved as MP4 files, which can be used for off-line analysis.<br>2. in vivo Calcium (GCaMP6) imaging in mice were collected with a fiber photometry apparatus, controlled by a software provided by the manufacturer, Thinkertech (Nanjing, China).<br>3. Brain slice electrophysiological data were collected with a path-clamp recording system that consists of Axon MultiClamp 700B amplifier, Digidata 1522B, and Clampex 10.7 software (Molecular Devices), etc.<br>4. Fluorescence imaging data were collected with a Zeiss LSM 880 confocal microscope and Zen2 software.
Data analysis	1. Locomotion behavioral data were analyzed with Ethovision (Noldus) and exported as Microsoft Excel files. The summary of data were copied into GraphPad Prism 7.0 for data illustration and statistical analysis.<br>2. Electrophysiological data were analyzed with Clampfit 10.7 (Molecular Devices), summarized with Microsoft Excel, and compared with GraphPad Prism 7.0 between or among groups.<br>3. Fiber photometry data were converted into Microsoft Excel files, and then converted into Clampfit files. Clampfit 10.7 was used to perform cross-correlation analysis between in vivo calcium signal.<br>4. Immunofluorescence data were analyzed with Fiji-ImageJ.

For manuscripts utilizing custom algorithms or software that are central to the research but not yet described in published literature, software must be made available to editors and reviewers. We strongly encourage code deposition in a community repository (e.g. GitHub). See the Nature Portfolio guidelines for submitting code & software for further information.

Data

Policy information about availability of data

All manuscripts must include a data availability statement. This statement should provide the following information, where applicable:

- Accession codes, unique identifiers, or web links for publicly available datasets
- A description of any restrictions on data availability
- For clinical datasets or third party data, please ensure that the statement adheres to our policy

All data in the main text and the supplementary materials are available from the corresponding authors upon request. Source data for Figs. 1 – 10 and Supplementary Figs. 1–11 are provided in this paper.

Field-specific reporting

Please select the one below that is the best fit for your research. If you are not sure, read the appropriate sections before making your selection.

☒ Life sciences ☐ Behavioural & social sciences ☐ Ecological, evolutionary & environmental sciences

For a reference copy of the document with all sections, see nature.com/documents/nr-reporting-summary-flat.pdf

Life sciences study design

All studies must disclose on these points even when the disclosure is negative.

Sample size	The number of mice used in each experiment was calculated in a priori power analysis (StatMate 2.0), the power of each experiment was set to 0.8.
Data exclusions	We confirmed the viral expression and location of the tips of optical fibers in mice after behavioral tests, and excluded data from mice with mis-targeted viral injection and expression, and optical fiber implants.
Replication	We let 2 performers test the same groups of mice separately. The attempts at replication were successful based on at least two independent experiments. behavioral experiments were performed at least twice in individual mouse more than 1 week apart.
Randomization	We randomly assigned 26-28 g mice (3-4 months old) into different groups for all experiments.
Blinding	1. For pain like behavioral tests, animal group information was withheld until after the experiment was completed.<br>2. For open field test and CPP test, the procedure and data analysis were made automatic with Ethovision. Therefore, no personal interference was introduced into data collection and analysis.<br>3. For electrophysiological recordings, the performers did not know whether the mice were from sham or SNI group, and the treatment of the mice were decoded after data analysis was completed.<br>4. For histological experiments, immunostaining and cell counting was conducted by two different performers.

Reporting for specific materials, systems and methods

We require information from authors about some types of materials, experimental systems and methods used in many studies. Here, indicate whether each material, system or method listed is relevant to your study. If you are not sure if a list item applies to your research, read the appropriate section before selecting a response.

Materials & experimental systems

n/a	Involved in the study	
☐	☒	Antibodies
☒	☐	Eukaryotic cell lines
☒	☐	Palaeontology and archaeology
☐	☒	Animals and other organisms
☒	☐	Human research participants
☒	☐	Clinical data
☒	☐	Dual use research of concern

Methods

n/a	Involved in the study	
☒	☐	ChIP-seq
☒	☐	Flow cytometry
☒	☐	MRI-based neuroimaging

Antibodies

Antibodies used	1. Primary antibody: c-Fos Rabbit mAb, #2250, Cell Signaling Technology; Guinea pig anti-NeuN, ,ABN90, Millipore; Mouse anti-CaMKII IgG, #50049, Cell Signaling Technology.<br>2. Secondary antibody. Donkey anti-rabbit IgG conjugated with Cy3 (code 711-165-152) or Donkey anti-rabbit IgG conjugated with Alexa Fluor 647 (code 711-605-152) , Jackson ImmunoResearch.
Validation	All Antibodies in the study are applicable on frozen section of rodent brain and have been validated in previous literatures.<br>1. Luan Y, et al. Reversal of hyperactive subthalamic circuits differentially mitigates pain hypersensitivity phenotypes in parkinsonian mice. Proc Natl Acad Sci U S A 117, 10045-10054 (2020).<br>2. Lacoste B, et al. Sensory-related neural activity regulates the structure of vascular networks in the cerebral cortex. Neuron 83, 1117-1130 (2014).<br>3. Singh A, et al. Mapping Cortical Integration of Sensory and Affective Pain Pathways. Current biology : CB 30, 1703-1715 e1705 (2020).

Animals and other organisms

Policy information about studies involving animals; ARRIVE guidelines recommended for reporting animal research

Laboratory animals	We used C57BL6 mice, Vgat-IRES-Cre (Slc32a1tm2(cre)Lowl/J) and Vglut2-IRES-Cre (Slc17a6tm2(cre)Lowl/J) transgenic mice (male, 3-4 months old) for all experiments. Mice were housed (no more than 5 per cage) on a 12-hour light/dark cycle (7:00 - 19:00) with ad libitum access to water and food. The animal room was controlled at a temperature of 23 °.
Wild animals	No wild animals was used in the study.
Field-collected samples	No field-collected sample was used the study.
Ethics oversight	The care and use of animals and the experimental protocols used in this study were approved by the Institutional Animal Care and Use Committee and the Office of Laboratory Animal Resources of the Xuzhou Medical University.

Note that full information on the approval of the study protocol must also be provided in the manuscript.
